# Tumour area infiltration and cell count in endoscopic biopsies of therapy-naive upper GI tract carcinomas by QuPath analysis: implications for predictive biomarker testing

**DOI:** 10.1038/s41598-023-43903-3

**Published:** 2023-10-16

**Authors:** Andreas H. Scheel, Hannah Lamberty, Yuri Tolkach, Florian Gebauer, Birgid Schoemig-Markiefka, Thomas Zander, Reinhard Buettner, Josef Rueschoff, Christiane Josephine Bruns, Wolfgang Schroeder, Alexander Quaas

**Affiliations:** 1 Institute of Pathology, University Hospital Cologne, Medical Faculty, University of Cologne, Kerpener Str. 62, 50937 Cologne, Germany; 2 Department of General, Visceral and Cancer Surgery, University Hospital Cologne, Medical Faculty, University of Cologne, Cologne, Germany; 3 Department of Internal Medicine I, University Hospital Cologne, Medical Faculty, University of Cologne, Cologne, Germany; 4 Targos Molecular Pathology GmbH, Kassel, Germany

**Keywords:** Targeted therapies, Cancer imaging

## Abstract

Guidelines regulate how many (tumour-bearing) tissue particles should be sampled during gastric cancer biopsy to obtain representative results in predictive biomarker testing. Little is known about how well these guidelines are applied, how the number of tissue particles correlates with the actual tumour-infiltrated area and how many absolute tumour cells are captured. The study included endoscopic biopsies of untreated carcinomas of the upper gastrointestinal (GI)-tract during the 2016–2020 review period. Archival (H&E)-stained histological sections were digitised and the tumour areas were manually annotated. The tumour-bearing tissue area and absolute carcinoma cell count per case were determined by image analysis and compared with a reference primary surgical specimen. Biopsies from 253 patients were analysed. The following mean values were determined: (a) tumour tissue particle number: 6.5 (range: 1–25, standard deviation (SD) = 3.33), (b) number of tumour-bearing tissue particles: 4.7 (range: 1–20, SD = 2.80), (c) tumour-infiltrated area: 7.5 mm^2^ (range: 0.18–59.46 mm^2^, SD = 6.67 mm^2^), (d) absolute tumour cell count: 13,492 (range: 193–92,834, SD = 14,185) and (e) tumour cell count in a primary surgical specimen (tumour size: 6.7 cm): 105,200,176. The guideline-recommended tissue particle count of 10 was not achieved in 208 patients (82.2%) and the required tumour-bearing tissue particle count of 5 was not achieved in 133 patients (52.6%). Tissue particle count, tumour-infiltrated area and tumour cell count were only weakly correlated. Most cases featured an infiltrated area ≥ 4.5 mm^2^ (156, 61.7%). Cases with more tissue particles showed only a moderate increase in infiltrated area and tumour cells compared to cases with fewer particles. Biopsies are often used to determine predictive biomarkers, particularly Her2/neu and PD-L1. Diagnostic standards to ensure representative material have been suggested in guidelines to reduce false-negative predictions. However, the real-world practice seems to substantially deviate from recommended standards. To the best of our knowledge, this is the first systematic study describing the relationships between endoscopic tissue fragment number, actual infiltrated tumour area and carcinoma cell number. The data question the tissue particle number as a quality assessment parameter. We advocate histopathological reports indicating on which basis statements on therapy-relevant biomarkers were made. Digital pathology has the potential to objectively quantify the tissue for documentation, quality assessment and future clinical studies.

## Introduction

Endoscopically-obtained tumour biopsies confirm the histological tumour identity but are also used to determine therapy-relevant biomarkers. In the case of inoperable patients or metastases that are difficult to access, these biopsies are the only tumour cell source available. Currently, clinically relevant predictive biomarkers in upper gastrointestinal tract carcinomas are Her2/neu and PD-L1. Future indications for targeted therapy are likely to expand the biomarker portfolio, possibly including Claudin 18.2 and FGFR2^1–4^. Gastric carcinomas can be divided into two anatomical regions: (a) proximal carcinoma of the cardia region, which may include adenocarcinomas of the gastroesophageal junction and (b) distal gastric carcinoma of the antrum region. The relevance of these tumour localisations are subject to significant geographic differences:

- in Eastern Europe, parts of Asia, South America: 80% distal (antrum)
- in Northern Europe and USA: 60% proximal (cardia/fundus)^5–7^.

In absolute numbers, an incidence of about 14,000 new cases per year is found in Germany (http://www.krebsdaten.de/magenkrebs). The proportion of cardiac carcinomas has been increasing in recent years.

Studies have determined how many endoscopic tissue particles are required to measure these biomarkers with high sensitivity^8–10^. Both Her2/neu and PD-L1 may exhibit heterogeneous expression. Accordingly, national guidelines state that "at least 8 particles should be taken from all suspicious lesions" and "a minimum of 10 particles is indicated in patients with large lesions" (German S3-guideline "Diagnosis and therapy of adenocarcinomas of the stomach and esophagogastric junction", long version). A German expert opinion on Her2/neu testing states more precisely that "…for reliable diagnosis of Her2/neu status the specific number of particles is less relevant than the quality of biopsies and the number of actual tumour-containing particles obtained" and calls for five tumour-containing particles from different tumour areas to avoid false-negative results due to the high intra-tumoral heterogeneity of Her2/neu^11^. Her2/neu stratification followed the known criteria of the TOGA trail. An adenocarcinoma of the upper GI tract is considered Her2/neu positive if (a) at least five contiguous carcinoma cells are Her2/neu positive on endoscopic biopsies or (b) at least 10% of the tumour is Her2/neu positive on surgical specimens^12^. PD-L1 is also a therapeutically relevant biomarker that is determined immunohistochemically on tumour tissue of carcinomas of the upper GI tract. The so-called combined positive score (CPS) is used for this purpose, which considers positivity on both tumour cells and certain mononuclear inflammatory cells (such as macrophages). As with Her2/neu, the endoscopically obtained tissue particles are often used for determination^2^.

In this study, we addressed the following questions: (A) how well are the requirements of the national S3 guideline are respected by gastroenterologists, oncologists and pathologists? (B) How well does the number of tissue particles correlate with the absolute area infiltrated by tumour and the absolute tumour cell count? Background: currently, there is no definition of what constitutes a “tumour-bearing biopsy particle”. Tumour-bearing particles could consist of only a few single tumour cells or be infiltrated entirely with tumour cells. The number of tumour-bearing particles would be the same, but the absolute area invaded by tumour cells and the actual tumour cell count would differ significantly in these two scenarios. With this premise, tumour area and absolute tumour cell count might be more objective and meaningful representative metrics than tumour-bearing particle count. (C) How many carcinoma cells are obtained by biopsies? (D) How many carcinoma cells make up a primarily operated adenocarcinoma of the oesophagus?

To answer these questions, (H&E)-stained histological sections were digitised. The number of tissue particles was determined and tumour-bearing area and absolute carcinoma cell count per case were assessed by image analysis.

To the best of our knowledge, this is the first such work on carcinomas of the oesophagus and stomach.

## Methods and material

### Specimen collection

**Figure 1:**
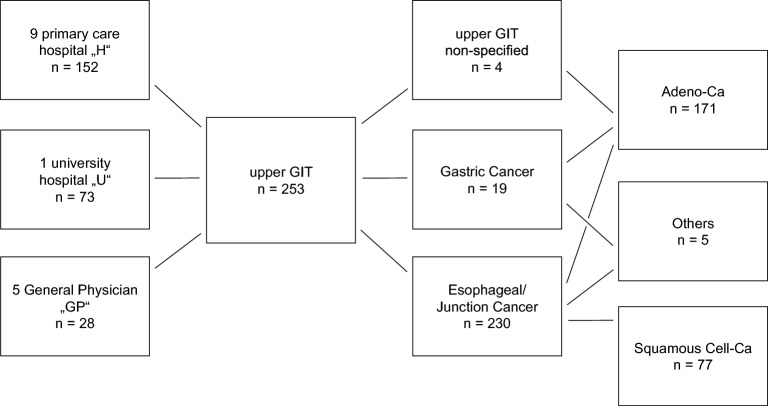
The tumor collective available for analysis is shown. It consists of a total of 253 patients. Biopsies were obtained endoscopically in a university hospital (U), peripheral hospitals (H) and gastroenterologists in private practice (GP). Biopsies of adenocarcinomas (n = 171) and esophageal tumors/transitional carcinomas (n = 230) are found quite predominantly.

We searched the pathology medical record system for all endoscopic tumour biopsies obtained from primary carcinomas of the oesophagus and stomach over a 6-year period (2016–2020). The biopsies were submitted by one university hospital (University Hospital Cologne), nine peripheral hospitals and five gastroenterologists (Fig. 1, Supplementary Fig. 1). All biopsies were processed and haematoxylin–eosin (H&E) stained in one histopathological laboratory according to established standard operating procedures (Institute of Pathology, University Hospital Cologne). Lymphomas or other histological tumour entities were excluded. The number of tissue particles taken was retrieved from the histopathological report and was microscopically re-evaluated. In the case of ambiguous wording in the written report such as "multiple particles", the particle number was determined microscopically. If there was a discrepancy of < 2 in the number of particles between the original report and microscopic evaluation during the course of the study, the information from the report was used, as a larger particle might have disintegrated during tissue processing. In the case of discrepancies of > 2 particles, which occurred only in a few cases, it was assumed that the number was primarily incorrectly evaluated and the number of particles from the microscopic evaluation was used for further analysis.

### Digitisation

The H&E-stained histological slides were digitised to whole-slide images using a NanoZoomer S360 whole-slide scanner (Hamamatsu Photonics, Japan; magnification 400×, mpp 0.2305). All cases in which the primary slides were not suitable for digitisation due to scratches or other artifacts were re-cut and re-stained from the original paraffin blocks (2 μm sections), with subsequent digitisation. Examples of histology and tissue particle numbers are shown in the supplementary data (Supplementary Fig. 2).

### Image analysis approach

The digitised slides were further processed using QuPath software (version 0.3.2). QuPath is a commonly used open-source software for digital pathology image analysis^13^.

**Figure 2:**
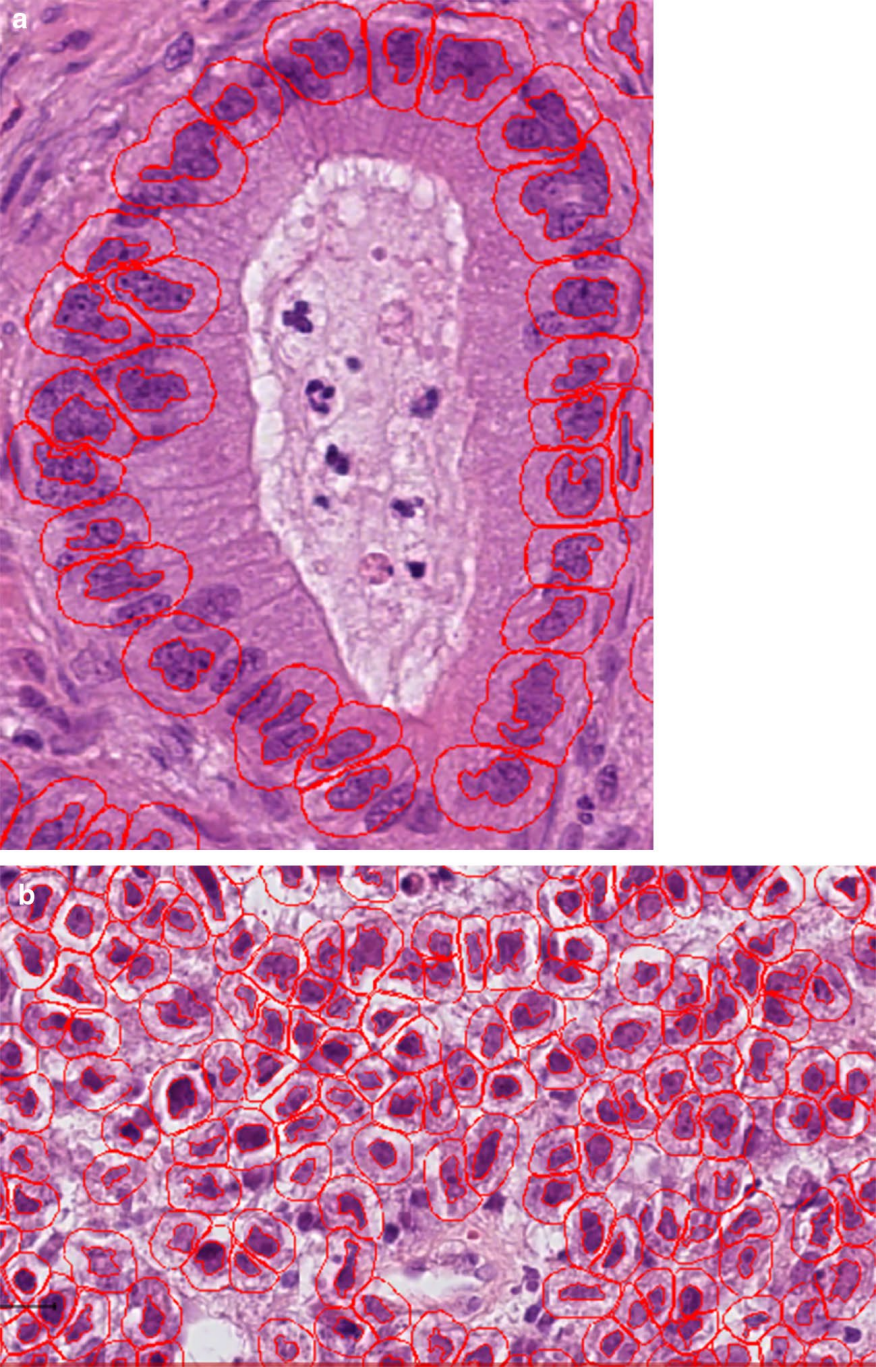
(**A**,**B**) Software-based detection of carcinoma cells: Qu-Path (version 0.3.2) marks the cells interpreted by the software as carcinoma cells. Surrounding stromal cells are not marked. A control by morphologically trained personnel/pathologists per case is therefore possible and reasonable.

The tumour-bearing areas in single tissue particles were manually annotated (HL and AQ). The absolute tissue area interspersed by tumour cells per case could thus be precisely quantified (mm^2^). For tumour cell detection, we initially tested two tools: a native cell detection tool from QuPath and a QuPath-implementation of StarDist algorithm^14^. The former provided visually better carcinoma cell detection (estimated by GI pathology expert, AQ) and allowed better flexibility concerning the parameter selection and was used for all further experiments. The main parameter change for cell detection tool defaults was minimum area of tumour nuclei, which was initially set at 25 µm^2^. Previously annotated tumour-bearing regions were processed by a cell detection tool to calculate the absolute tumour cell numbers (Fig. 2A,B). For this, detection results for all regions were morphologically controlled by two human analysts (HL and AQ). If cell detection results were suboptimal (primarily through “contamination” by stromal cells), the minimum nuclei area was adjusted (range: 25–40 µm^2^), allowing the adequate separation of tumour cells from other cell populations in virtually all cases (Fig. 2). In single cases, tumour cell detection was still not optimal, mostly as consequence of artificial changes and local cutting/staining artifacts. In such cases, the carcinoma cell numbers were manually counted (HL and AQ).

### Quantifying tumour cell number on a reference surgical specimen

A representative case of the tubular (intestinal) differentiated adenocarcinoma of the oesophagus treated with primary surgery was selected. The specimen was routinely processed according to the institute standards and the tumour was completely submitted for paraffin embedding (n blocks = 29) The largest tumour dimension was 6.7 cm. One representative paraffin block was completely cut in 50 μm steps (thickness 2 µm) yielding 50 histological sections. Since an average tumour cell in this case had a diameter of 25–30 μm, the stepwise sequence of 50 μm ensured that a new tumour cell layer was present on each new H&E step section. The tumour area of the remaining paraffin blocks was used to calculate the absolute tumour cell numbers considering three dimensions of the respective tissue fragments. All H&E-stained step sections (29 + 50 = 79) were digitised and tumour cell number calculated using QuPath, as detailed above.

### Statistics

Data were recorded in Microsoft Excel (Version 2016). Statistical analyses were performed using R statistical programming language (version 4.1.2) including the packages ggplot2 and patchwork. For statistical testing, Student's T-test was used. Alpha was set to 5% and corrected for the multiple comparisons problem with the Bonferroni method (α/[number of tested hypotheses]). For subgroup analyses, the respective subgroup was tested against the rest of the collective.

### Ethics declaration

Patients gave their written consent to usage of their tumor specimens. The objective of the project is primarily in the field of diagnostics and quality assurance, an approval was obtained from the University of Cologne Ethics Committee. An approval was obtained from the University of Cologne Ethics Committee (reference number: 20-1583 and 10-242).

All authors confirm that methods used were carried out in accordance with relevant guidelines and regulations. The experimental protocols were approved by the licensing committees. We confirm that informed consent was obtained from all subjects and/or their legal guardians.

## Results

### Baseline characteristics

In the considered period of 2016–2020, 253 patient cases with endoscopically obtained biopsies of primary carcinomas of the upper gastrointestinal tract were available. These included 230 oesophageal carcinomas, 19 gastric carcinomas and 4 carcinomas spanning both regions. Histologically, 171 (67.6%) were adenocarcinomas including 143 tubular-intestinal carcinomas, 9 poorly cohesive carcinomas including signet ring cell carcinoma, 16 well differentiated carcinomas and 3 tumours showing mucinous differentiation, while 77 were squamous cell carcinomas (30.4%) and 5 were other subtypes (2%). Most patients were male (76.7%) (Supplementary Table 1).

The biopsies were submitted either by small primary care hospitals ('H', n = 9 hospitals, n = 152 cases), the university hospital ('U', n = 73 cases) or by general physician practices ('GP', n = 5 physicians, n = 28 cases) (Table 1; Fig. 1; Supplementary Fig. 1).

**Table 1:** Results of analyzing n = 253 biopsy cases.

		All	Submitting Institution			Histotype		
			U	H	GP	Adeno	Squamous	Other
Cases (percent of all)		253	73 (28.9%)	152 (60.1%)	28 (11.1%)	171 (67.6%)	77 (30.4%)	5 (2%)
Per case, mean (± SD)	Tissue particles	6.5 (± 3.3)	6.33 (± 2.85)	6.72 (± 3.32)	5.86 (± 4.39)	6.36 (± 3.2)	6.65 (± 3.13)	9.4 (± 8.4)
	Tumor-bearing tissue particles	4.68 (± 2.8)	4.45 (± 2.29)	4.85 (± 2.87)	4.36 (± 3.57)	4.53 (± 2.55)	4.88 (± 2.77)	6.8 (± 8.04)
	Tumor-infiltrated area (tiA)	7.46 (± 6.67)	7.96 (± 6.00)	7.48 (± 7.22)	6.07 (± 4.91)	6.85 (± 5.1)	8.95 (± 9.15)	5.51 (± 6.29)
	Tumor cell count (tcN)	13,492 (± 14,185)	15,116 (± 15,012)	12,954 (± 13,835)	12,175 (± 13,996)	12,860 (± 14,326)	15,267 (± 14,060)	7763 (± 8884)

Arithmetic mean and standard deviation of the respective parameters are listed. The results are subdivided by the submitting institutions and by histotype (U, university hospital; H, peripheral hospitals; GP, gastroenterologists. Adeno, adenocarcinomas; squamous, squamous cell carcinomas; other, other histotypes). No significant differences were detected among the three subgroups.

### Total number of tissue particles

**Figure 3:**
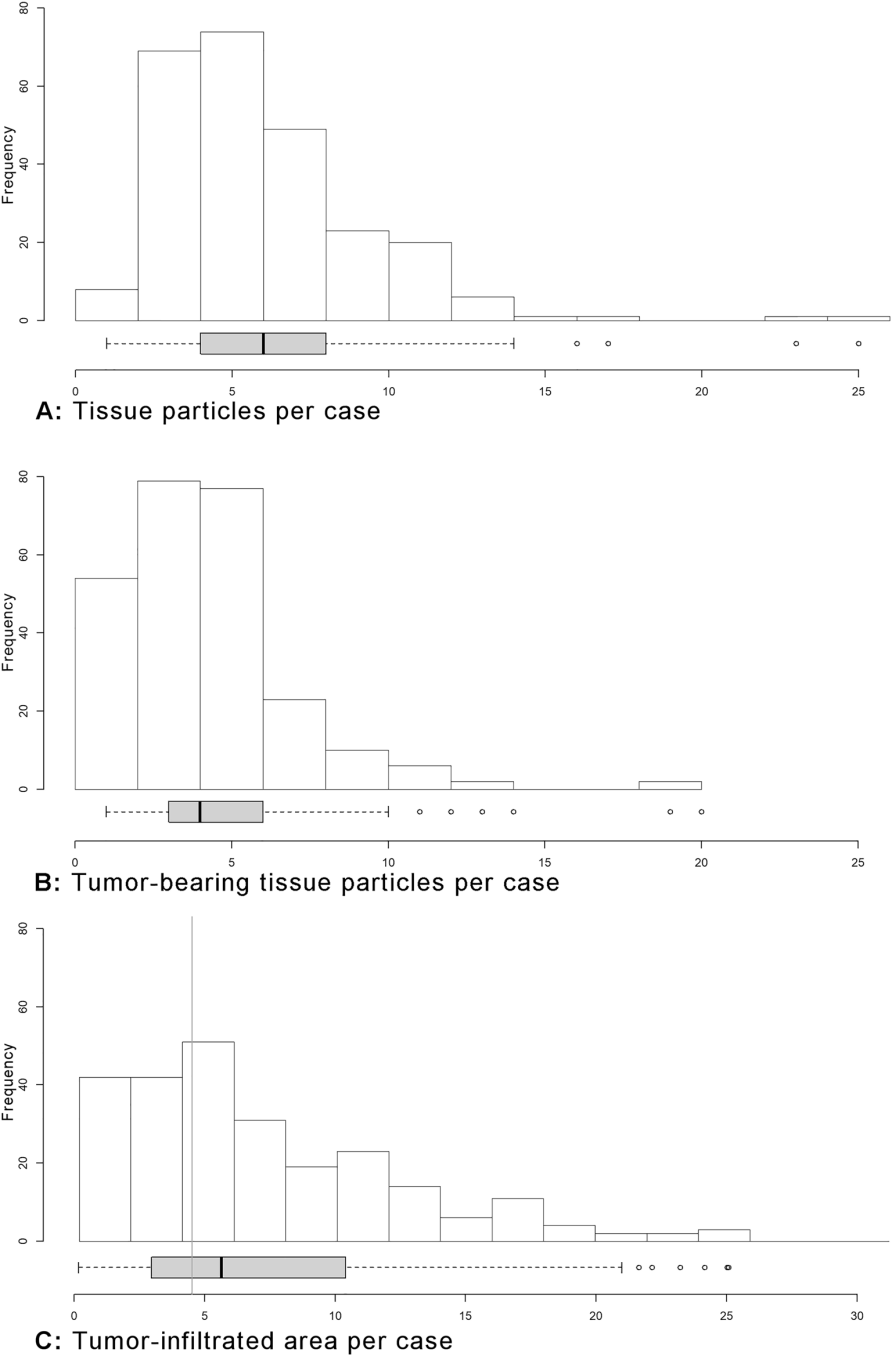
Distributions of the count of tissue fragments and tumor-infiltrated area, depicted as histograms and corresponding boxplots, (**A**) total count of tissue fragments per case, frequency of cases; (**B**) count of tumor-bearing tissue fragments per case. (**C**) Size of tumor-infiltrated area in mm^2^. Vertical line: 4.5 mm^2^.

On average, each case contained 6.5 tissue particles [range: 1–25, standard deviation (SD) = 3.33] (Table 1, Fig. 3). There were no significant differences between the three groups of submitting institutions (U, H and GP). Among the submitting institutions, one general physician had significantly fewer tissue particles per case ('GP1'; n = 17 cases, mean particles 4.35, p < 0.001). The number of cases and tissue particles per submitting institution are summarised in the supplementary data (Supplementary Fig. 1).

Subgroup-analysis by histotype and submitting institution showed no significant differences: Adenocarcinoma: n = 6.4 particles/per case (H = 6.7, U = 6.1, GP = 5.7) (1–25, SD = 3.20); squamous cell carcinoma: 6.9 particles/per case (H = 6.4, U = 7.3, GP = 7.0) (2–17, SD = 3.13) (p = 0.508).

Most cases did not achieve the recommended tissue particle numbers of n = 10 for major lesions and n = 8 for all indeterminate lesions, n < 10: 208 of 253 cases, 82.2%; n < 8: 174 of 253 cases, 68.8%. On the other hand, a tumour-infiltrated area (tiA) of ≥ 4.5 mm^2^ was achieved in 61.7% of cases (Fig. 3C).

### Number of tumour-bearing tissue particles

The mean number of tumour-bearing tissue particles was 4.68 per case (1–19, SD = 2.8) (Fig. 3B). Again, subgroup-analysis did not show any significant differences per histotype or submitting institution. Adenocarcinoma: mean = 4.4 (H = 4.75, U = 4.26, GP = 4.24) (1–19, SD = 2.55); squamous cell carcinoma: mean = 5.1 (H = 4.78, U = 5.11, GP = 5.33) (1–14, SD = 2.77) (p = 0.338). In 69 cases (27.3%), all particles were tumour-bearing, while half of the particles or more were tumour-bearing in 217 cases (86%). Overall, the ratio of tumour-bearing/all tissue particles was 73.3% (7.7–100%, SD = 23.7%).

The required number of tumour-bearing particles, n ≥ 5, was not achieved in 133 of 253 cases (52.6%).

Cases with n ≥ 10 particles and n ≥ 8 are enriched for n ≥ 5 tumour-bearing particles, 39/45, 86.7% and 68/78, 86.0%.

### Tumour cell count and infiltrated area

**Figure 4:**
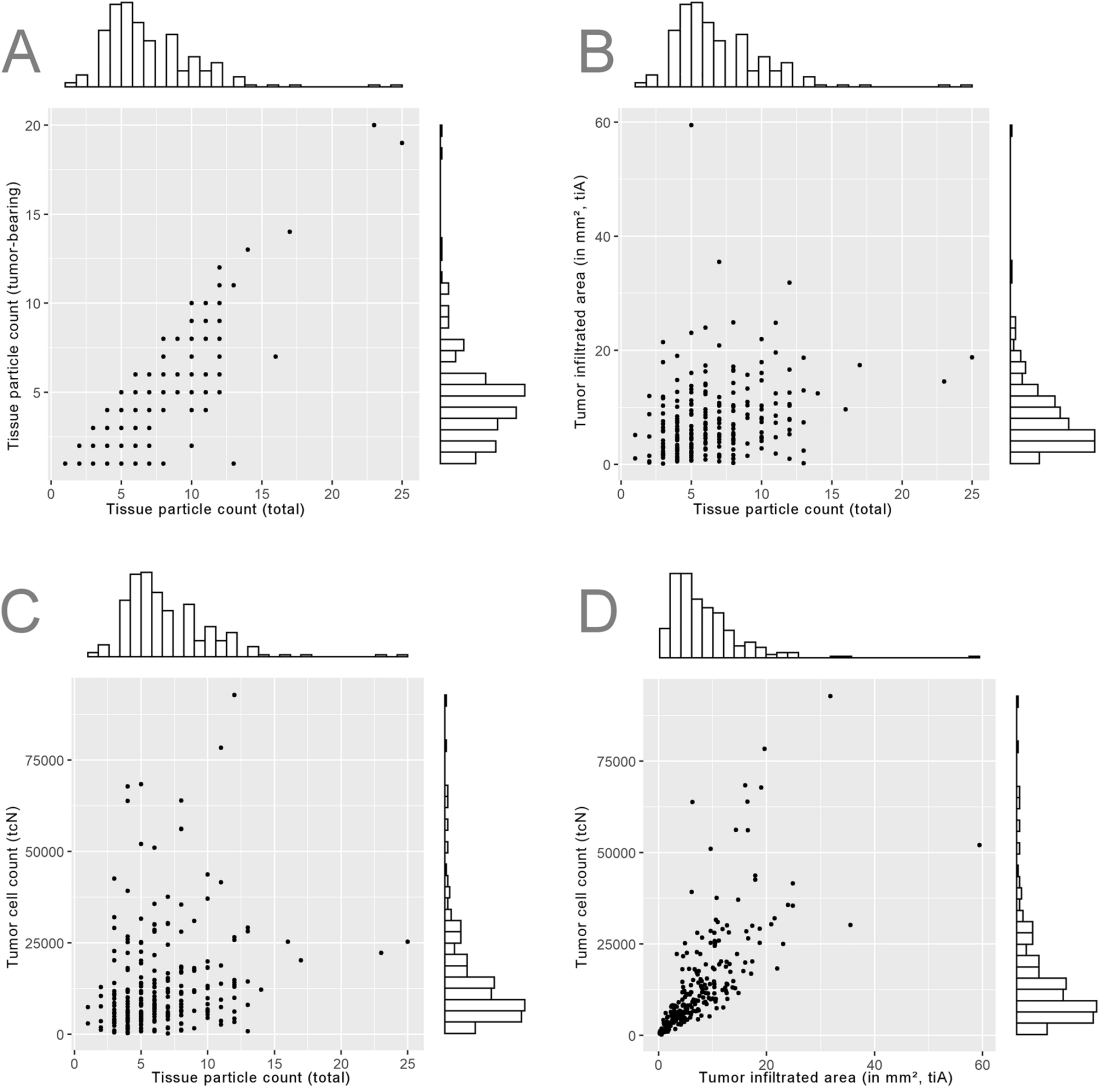
Relation of the tissue particle count, tumor cell count (tcN) and area infiltrated by tumor (tiA) represented by 2D-scatterplots with side-histograms of the respective distributions. (**A**) The total count of tissue particles and the count of tumor-bearing tissue particles are interrelated (tumor-bearing ≤ total count; right-hand spread of datapoints). (**B**,**C**) The count of tissue fragments is only weakly correlated to tiA (r = 0.28, upper right) and to tcN (r = 0.20, lower left). (**D**) tiA and tcN show a strong linear correlation (r = 0.71).

On average, 13,492 carcinoma cells were detected per case (SD = 14,185). The cell number was only weakly correlated with the total tissue particle count (r = 0.20) and the tumour-bearing tissue particle count (r = 0.38) (Fig. 4). Subgroup-analysis by histotype did not show significant differences, adenocarcinoma: mean = 12,860 (193–92,834, SD = 14,326), squamous cell carcinoma: mean = 15,267 (494–63,979, SD = 14,059) (p = 0.217). Cases with n ≥ 8 and n ≥ 10 tissue particles featured significantly more tumour cells, on average 17,233 vs. 11,793 (p = 0.0119) and 18,131 vs. 12,488 (p = 0.0510).

The average tiA per case was 7.46 mm^2^ (0.18–59.46 mm^2^, SD = 6.66 mm^2^). The tiA was substantially correlated with the tumour cell number (r = 0.71). Tissue particle count (r = 0.28) and tumour-bearing particle count (r = 0.46) showed low correlations.

The mean area per tissue particle was 1.29 mm^2^ (0.02–11.89 mm^2^, SD = 1.29mm^2^). The mean number of tumour cells per tumour-bearing particle was 3,033 (97–22,595, SD = 2988).

Subgroup-analysis by histotype showed a small but not significant difference: adenocarcinoma mean tiA = 6.85 mm^2^ (0.2–31.83 mm^2^, SD = 5.1 mm^2^); squamous cell carcinoma mean tiA = 8.95 mm^2^ (0.19–59.46 mm^2^, SD = 9.1 mm^2^) (p = 0.06191).

Cases with n ≥ 8 and n ≥ 10 tissue particles had a significantly higher tiA; 9.5 vs. 6.54mm^2^ (p < 0.001) and 10.54 vs. 6.8 mm^2^ (p = 0.001). However, cut-off analysis showed that cases with fewer particles had a relatively large tiA and tcN (Table 2), e.g. with ≥ 2 particles as cut-off, 61.8% of cases showed a tiA ≥ 4.5 mm^2^. Increasing the particle cut-off was associated with only moderate increases in tiA and tcN. Moreover, cases with fewer particles had a higher tcN count per particle compared to cases with more particles. A particle count ≥ 4 was achieved in 85.8% of cases, 64.5% of which had a tiA ≥ 4.5 mm^2^ (Table 2, Supplementary Table 2).

**Table 2:** Analysis of different particle count cut-offs (≥ 2 to ≥ 10).

		Cut-off (tissue particle count, n ≥ x)								
		10	9	8	7	6	5	4	3	2
Cases (% of all cases)		45 (17.8%)	53 (20.9%)	79 (31.2%)	102 (40.3%)	134 (53%)	176 (69.6%)	217 (85.8%)	245 (96.8%)	251 (99.2%)
Cases tiA ≥ 4.5mm^2^ (% of fraction)		39 (86.7%)	45 (84.9%)	62 (78.5%)	76 (74.5%)	99 (73.9%)	120 (68.2%)	140 (64.5%)	152 (62%)	155 (61.8%)
Per case, mean (± SD)	Tissue particles	12 (± 3)	11.6 (± 3)	10.4 (± 3)	9.6 (± 3)	8.8 (± 3)	7.9 (± 3.1)	7.1 (± 3.2)	6.7 (± 3.3)	6.6 (± 3.3)
	Tumor-bearing tissue particles	8.2 (± 3.8)	7.9 (± 3.6)	7.2 (± 3.3)	6.6 (± 3.2)	6.2 (± 2.9)	5.6 (± 2.8)	5.1 (± 2.8)	4.8 (± 2.8)	4.7 (± 2.8)
	Tumor-infiltrated area (tiA)	10.5 (± 6.6)	10.4 (± 6.4)	9.5 (± 6.3)	9.1 (± 6.6)	8.8 (± 6.3)	8.5 (± 7.2)	7.8 (± 6.9)	7.6 (± 6.7)	7.5 (± 6.7)
	Tumor cell count (tcN)	18,131 (± 17,973)	17,573 (± 16,902)	17,234 (± 16,986)	16,085 (± 15,788)	15,590 (± 14,902)	14,768 (± 14,591)	14,241 (± 14,700)	13,738 (± 14,330)	13,558 (± 14,220)
	Tumor cell count per tumor-bearing particle	2242 (± 1944)	2244 (± 1834)	2350 (± 1977)	2408 (± 1914)	2484 (± 1965)	2621 (± 2151)	2852 (± 2769)	2981 (± 2948)	3016 (± 2987)

Since the counts of all tissue particles per case and the count of tumor-bearing particles per case are both dependent and positive correlated, a higher cut-off is associated with more tumor-bearing particles. The tumor-infiltrated area (tiA) and the tumor cell count (tcN) are only weakly correlated to the tissue particle count. A higher cut-off is associated only with an low increase in tiA and tcN. Interestingly, the count of tumor cells per tumor-bearing particle even decreases with higher cut-offs.

The reference adenocarcinoma of the oesophagus with the largest diameter of 6.7 cm contained 105,200,176 tumour cells, i.e. 105*10^6^.

## Discussion

In the present study, we measured the tissue particle number, tiA and tumour cell number (tcN) in a cohort of n = 253 tumour-biopsies of the upper GI-tract by digital image-analysis. Both tiA and tcN were only weakly correlated to the number tissue particles and the number of tumour-bearing particles. The particles showed a broad spectrum of different sizes (mean tissue area per case: 7.46 mm^2^, SD = 6.66 mm^2^; average particle size: 1.29 mm^2^, SD = 1.29 mm^2^). Subgroup-analyses of submitting institutions and histotypes did not show any relevant differences. Cut-off analysis showed that 17.8% of cases (45/253) contained ≥ 10 tissue particles and 85.8% contained ≥ 4 particles (217/253). A tiA ≥ 4.5 mm^2^ was achieved by 61.7% of all cases (156/253).

Effective personalised oncology relies on targeted drugs and patient stratification by predictive biomarkers. The first targeted drug approved in gastric carcinoma/carcinomas of the oesophago-gastric transition zone was the anti-Her2-antibody trastuzumab^1^. Her2-testing is often performed on biopsy material and heterogeneous expression is an important issue^15–17^. Thus, a group of experts suggested the collection of five tumour-bearing tissue particles from different tumour areas to avoid false-negative results^11^.

Current guidelines focus on the number of tissue particles that should be obtained. However, from our perspective, a histology-based definition of what constitutes a 'tissue particle' has not been established. Depending on the endoscopy device used, particles may vary in size. The tissue may be completely interspersed with tumour or may only marginally harbour individual tumour cells. Tumour-necrosis may be present and reduce the number of vital carcinoma cells for analysis. In conclusion, the number of tissue particles seems less relevant for the determination of biomarkers compared to the overall tissue quality, i.e. the number and area infiltrated of vital tumour cells. The number of tissue particles can only be a benchmark for endoscopists who biopsy a tumour. Histopathologists, who determine predictive biomarkers based on these tissue particles, should be more precise about the actual quality. A negative biomarker result may be a contraindication for a potentially life-prolonging therapy option. Biopsy material with insufficient carcinoma cells should not be tested and the collection of additional tumour material should be discussed^11^.

The aim of this study was to increase the awareness of all involved disciplines to this factual problem.

In the analysed patient cohort, the biopsy requirements of the German S3 guideline were not met in every case. In 208 patients (82.8%), < 10 tissue particles were taken, while < 5 tumour-bearing particles were detected in 133 patients (52.6%). Thus, the reasons are speculative: (a) ignorance of the S3 guideline, (b) a fear of adverse-effects, especially bleeding and (c) estimation to have identified enough tumour-bearing tissue. It must be noted that this is a study of a metropolitan region in Western Germany and can in no way claim to be representative of the whole country. A multicentre study of different regions and federal states would be necessary and, in our opinion, would also be of interest.

The employed digital-image analysis to quantify tiA and tcN is easy to apply. The QuPath software is open-source and free of charge. It is compatible with all common histological whole-slide scanners. We suggest that the quantitation of tissue particle numbers and tumour cell content should be included in studies of histological biomarkers to verify representativeness. Still, the procedure has yet to be optimised and validated. However, we have deliberately chosen the freely available software QuPath, since all working groups worldwide are given the opportunity to carry out similar investigations.

Overall, 83% of the investigated adenocarcinomas showed tubular (intestinal) tumour differentiation, while 4% corresponded to poorly cohesive carcinoma including signet ring cell carcinoma. Different subtypes of carcinoma are found in different parts of the world due to the varying risk constellations. In the East-Asian region, the proportion of Helicobacter-related and distally localised gastric carcinomas is much higher than in Western Europe, where this tumour type has been declining for years. This is also accompanied by the high proportion of tubular-intestinal differentiated tumours in Western Europe. This explains the high proportion of adenocarcinomas of the oesophagus and junction-tumours in our collective.

There were no significant differences between the submitting institutions if grouped into university hospital, primary care hospitals and general physicians. Still, there was obvious variation between the cases (Supplementary Fig. 1). One general physician submitted significantly fewer tissue particles. The range of biopsies per case was 1–25 (mean = 6.5). Overall, the variation in particle numbers seemed larger between the cases than between the institutions.

Our group previously analysed the relevance of heterogeneity of PD-L1 expression in gastric carcinoma and demonstrated that at least four tumour-bearing tissue particles with a total area of 4.5 mm^2^ are necessary to reach an acceptable predictive probability of the actual PD-L1 value^10^. In the present study, the mean area per case was 7.46 mm^2^ and a tiA of 4.5 mm^2^ was achieved by the majority of cases.

It has to be noted that the patient cohort included only patients with successful tumour biopsies, i.e. at least some histologically convincing tumour cells were present in each case. Subsequent studies could include cases with false-negative biopsies, as revealed by follow-up biopsies or surgery. This would allow positive and negative predictive values and their relation to tissue particle counts tiA and tcN to be investigated.

Our study has further limitations: The absolute quantification of carcinoma cells in routine H&E sections was adequate in most cases with the selected methodology and software tool (QuPath) with no or minimally acceptable levels of contamination through other cells. However, individual determination of the optimal software settings for tumour cell nuclei detection might be necessary, which in our case was between 25 and 40 μm^2^. This threshold and, therefore, the quantification accuracy in routine H&E sections might be dependent on pre-analytical factors including fixation, FFPE-preparation, cutting and staining quality as well as digitisation parameters. At present, all software for digital pathology require site-specific training, optimisation and validation.

During the course of manuscript development, the question arose how many tumour cells constitute an adenocarcinoma of the oesophagus (EAC) not treated with neoadjuvant therapy. We were able to show that a 6.7 cm large EAC consists of just over 100 million tumour cells (in addition to the extracellular matrix, inflammatory cells and capillaries). We also received biopsy-material from the same patient preoperatively. Three tissue particles contained a total of 11,009 carcinoma cells, which infiltrated an area of 7.67 mm^2^. Thus, the biopsy in this case accounted for 0.01%.

## Conclusion

This is the first work to systematically capture the relationships between endoscopically obtained tissue particle number, actual infiltrated tumour area and absolute carcinoma cell count. By considering absolute carcinoma cell count or tumour area, future clinical studies will be more standardised and comparable. We advocate that histopathological reports should indicate on which basis (tissue particle number or carcinoma cell number or tumour area) statements on therapy-relevant biomarkers are made. In the case of the negative detection of a predictive biomarker, it is highly relevant whether this statement is based on sufficiently representative tumour material.

### Supplementary Information

Supplementary Legends.

Supplementary Figure 1.

Supplementary Figure 2.

Supplementary Table 1.

Supplementary Table 2.

## Data Availability

The datasets generated during and/or analysed during the current study are available from the corresponding author on reasonable request.
