# Supplementary material for: Tumour area infiltration and cell count in endoscopic biopsies of therapy-naive upper GI tract carcinomas by QuPath analysis: implications for predictive biomarker testing
Source: Sci Rep. 2023 Oct 16;13:17580. doi: 10.1038/s41598-023-43903-3 (PMC10579338; doi:10.1038/s41598-023-43903-3)
Supplement: Supplementary file 1 — Supplementary Legends. [file 41598_2023_43903_MOESM1_ESM.docx]

**Supplementary Figure 1:** Submitting institutions, cases and tissue fragment numbers per case. Five general physicians (GP1 – GP5), nine primary care hospitals (H1 – H9) and one university hospital (U) submitted a total of n=253 cases. The boxplots show the tissue fragment numbers per case for each institution, n=number of submitted cases, mean=average number of tissue fragments per case, SD=standard deviation, p-value. There are no significant differences between the three groups GP, H and U. GP1 showed significantly fewer biopsies per case compared to all other institutions (p<0.001).

**Supplementary Figure 2:** Two cases showing representative histology and range of material. A1, A2: A case with n=12 biopsy particles, some fragmentation; total area 10.42mm2 , 12/12 particles infiltrated, tumor cell count 19237. B1, B2: A case with n=2 biopsy particles; total area 0.55mm2, 1/2 particles infiltrated, tumor cell count 302. A1, B1: Low magnification (10x), scale bar=2.5mm; A2, B2: High magnification (100x), scale bar=250µm.

**Supplementary Table 1:** Baseline characteristics of the analyzed cases.

**Supplementary Table 2:** Subanalysis of cases with ≥4 tissue particles.
