# Supplementary figures and images for: Tumour area infiltration and cell count in endoscopic biopsies of therapy-naive upper GI tract carcinomas by QuPath analysis: implications for predictive biomarker testing
Source: Sci Rep. 2023 Oct 16;13:17580. doi: 10.1038/s41598-023-43903-3 (PMC10579338; doi:10.1038/s41598-023-43903-3)

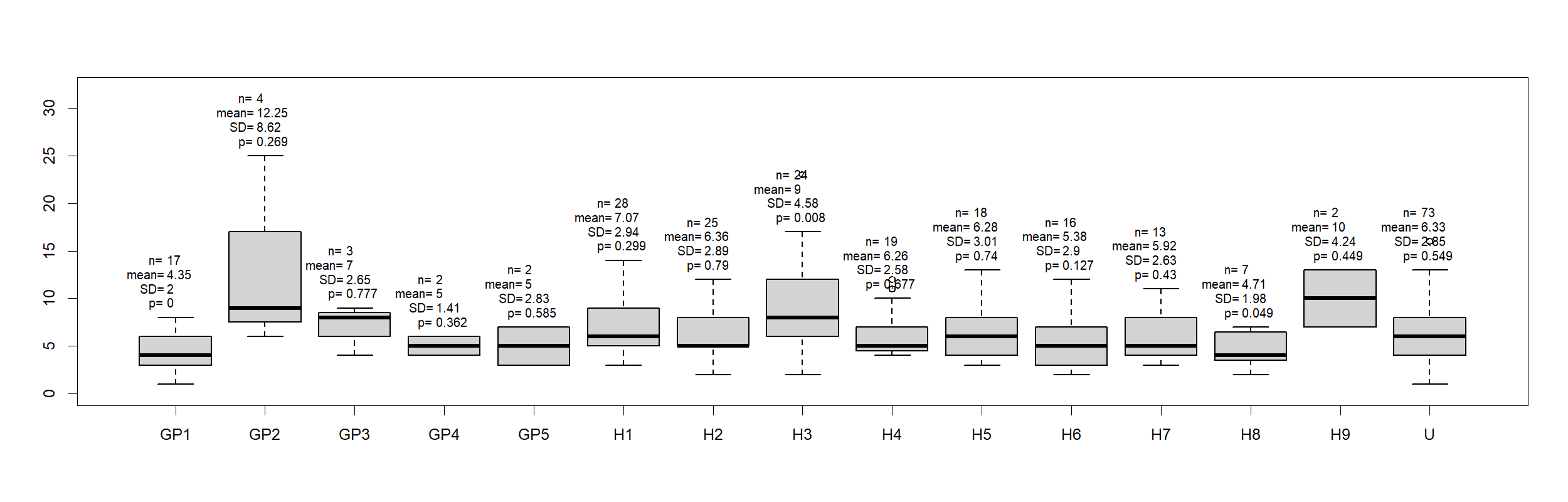

Supplement: Supplementary file 2 — Supplementary Figure 1. [file 41598_2023_43903_MOESM2_ESM.tiff]

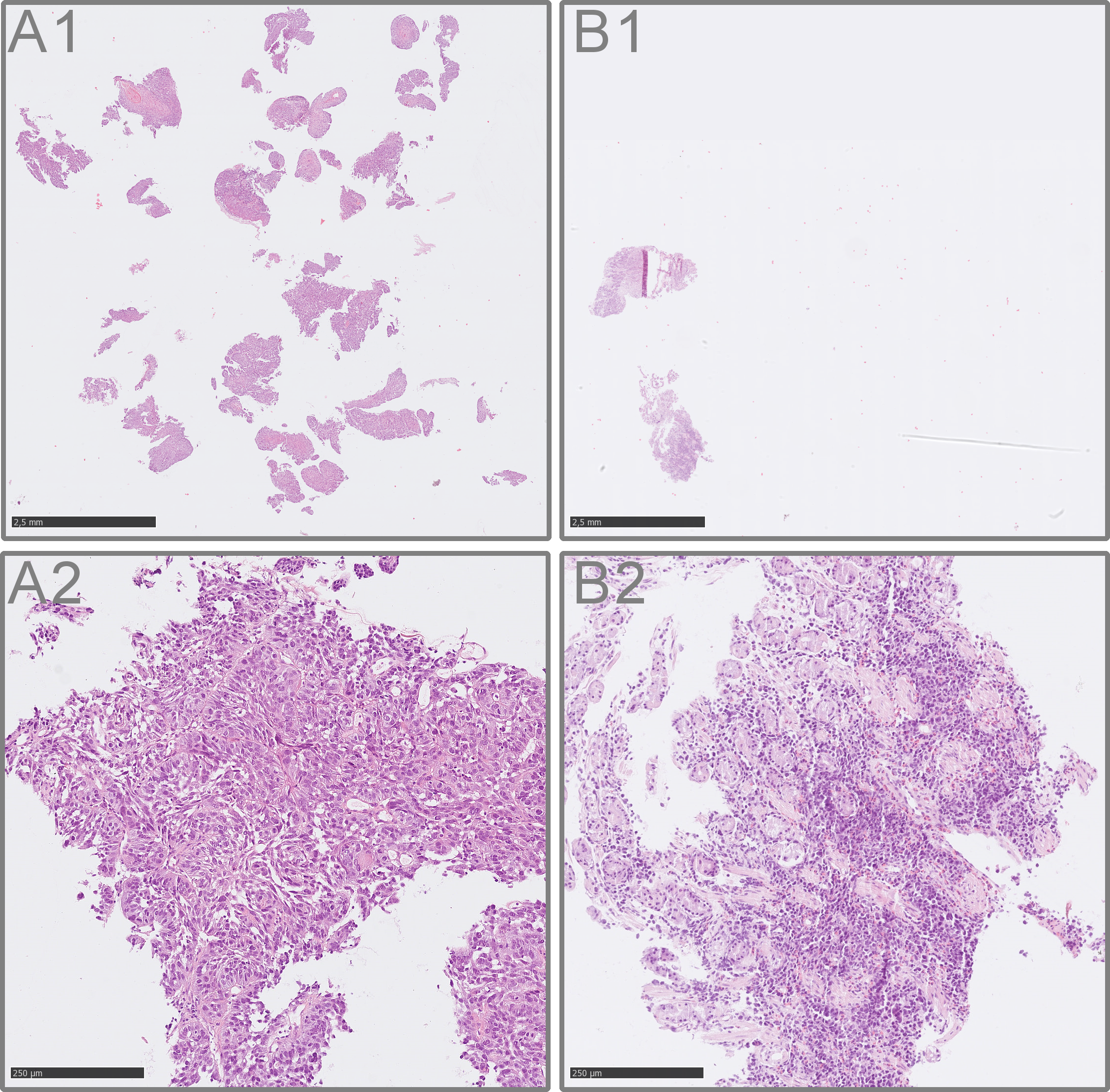

Supplement: Supplementary file 3 — Supplementary Figure 2. [file 41598_2023_43903_MOESM3_ESM.tif]

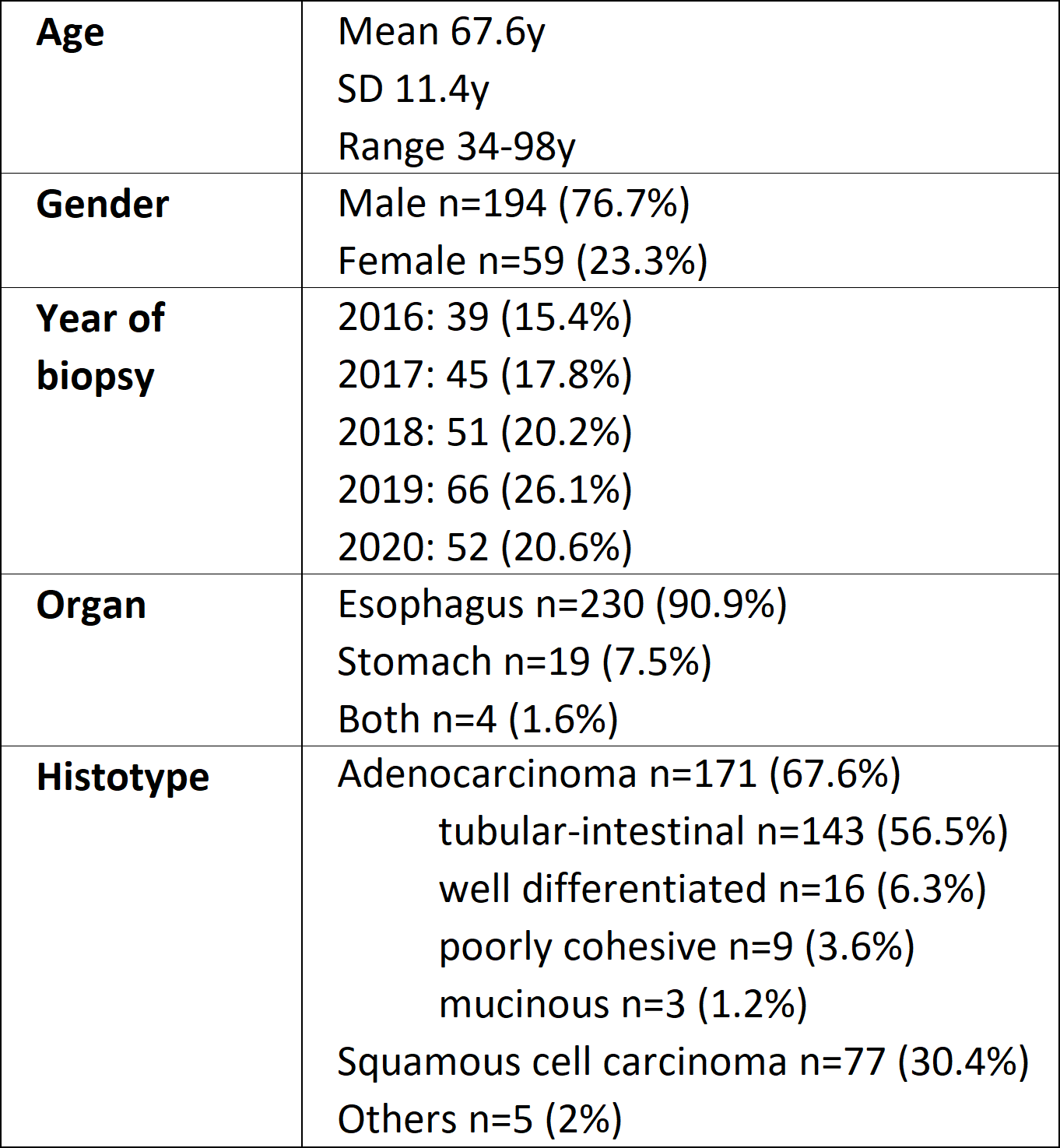

Supplement: Supplementary file 4 — Supplementary Table 1. [file 41598_2023_43903_MOESM4_ESM.tif]

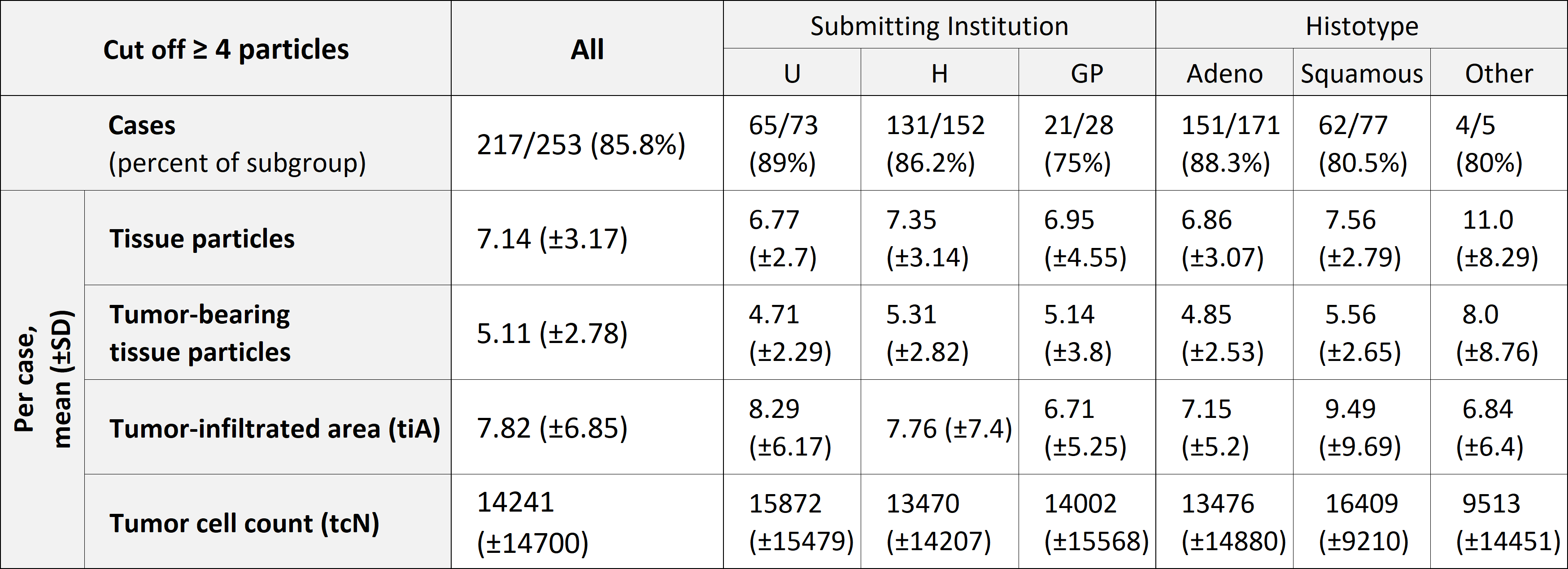

Supplement: Supplementary file 5 — Supplementary Table 2. [file 41598_2023_43903_MOESM5_ESM.tif]
